# Comparison of Host Cytokine Response in Piglets Infected With Toxigenic and Non-toxigenic *Staphylococcus hyicus*

**DOI:** 10.3389/fvets.2021.639141

**Published:** 2021-02-16

**Authors:** Yan Li, Hongchao Gou, Pinpin Chu, Kunli Zhang, Zhiyong Jiang, Rujian Cai, Shuai Song, Zhibiao Bian, Chunling Li

**Affiliations:** ^1^Institute of Animal Health, Guangdong Academy of Agricultural Sciences, Guangzhou, China; ^2^Guangdong Provincial Key Laboratory of Livestock Disease Prevention, Guangzhou, China; ^3^Guangdong Open Laboratory of Veterinary Public Health, Guangzhou, China; ^4^Scientific Observation and Experiment Station of Veterinary Drugs and Diagnostic Techniques of Guangdong Province, Guangzhou, China

**Keywords:** *Staphylococcus hyicus*, toxigenic and atoxigenic, cytokine response, interleukin-10, pathogenicity

## Abstract

*Staphylococcus hyicus* is the most common causative agent of exudative epidermitis (EE) in piglets. *Staphylococcus hyicus* can be grouped into toxigenic and non-toxigenic strains based on its ability to cause EE in pigs. However, the inflammatory response of piglets infected with toxigenic and non-toxigenic *S. hyicus* has not been elucidated. In this study, we evaluated the serum cytokine profile in piglets inoculated with toxigenic and non-toxigenic *S. hyicus* strains and recorded the clinical signs in piglets. Fifteen piglets were divided into three groups (*n* = 5) and inoculated with a toxigenic strain (ZC-4), a non-toxigenic strain (CF-1), and PBS (control), respectively. The changes in serum levels of cytokines (interleukin [IL]-1β, IL-4, IL-6, IL-8, IL-10, IL-12, granulocyte-macrophage colony-stimulating factor, interferon-γ, transforming growth factor-β1, and tumor necrosis factor-α) were evaluated using a cytokine array at 6, 24, 48, and 72 h post inoculation. The results showed that piglets infected with the toxigenic strain exhibited more severe clinical signs and higher mortality than those infected with the non-toxigenic strain. The serum levels of pro-inflammatory cytokine IL-1β were significantly increased in toxigenic-and non-toxigenic-strain-infected piglets compared to those in the control group (*p* < 0.05), while the anti-inflammatory cytokine IL-10 was significantly up-regulated only in toxigenic group than in control group (*p* < 0.05). These results indicated that piglets infected with toxigenic and non-toxigenic *S. hyicus* showed differential infection status and inflammatory responses. Both toxigenic- and non-toxigenic- *S. hyicus* infection could induce a pro-inflammatory reaction in piglets. In addition, the toxigenic strain induced a strong anti-inflammatory response in piglets as indicated by the increased serum level of IL-10, which may be associated with the severe clinical signs and increased mortality and may be the key cytokine response responsible for pathogenic mechanisms of *S. hyicus*.

## Introduction

*Staphylococcus hyicus*, a gram-positive bacterium, is one of the major opportunistic and zoonotic pathogens causing exudative epidermitis (EE) in pigs, primarily suckling and newly weaned piglets (1, 2). Exudative epidermitis is characterized by exfoliation of the skin; a thick, greasy, brown greasy exudate (3); suppurative pneumonia; and sepsis (4), which may lead to dehydration and subsequent death (5). Remarkably, a strain of *S. hyicus* was isolated from the blood culture and bone from a man suffering from debilitating subacute lumbar pain (6). Previous studies indicate that exfoliative toxin is the main virulence factor that induces the disease (7, 8). According to its ability to induce EE and to produce exfoliative toxin, *S. hyicus* has been divided into toxigenic and non-toxigenic strains (8, 9). At least five exfoliative toxins, named SHETB (10), ExhA, ExhB, ExhC, and ExhD (11) have been described. The exfoliative toxins selectively digested porcine desmoglein 1 directly in the porcine epidermis, causing separation of cells in the stratum spinosum and rapid intraepidermal spread of organisms (12).

Cytokines are a group of small, secretory proteins that mediate a variety of immunomodulatory and inflammatory responses (13, 14). It is well-known that inflammatory cytokines, including pro-inflammatory cytokines [e.g., interleukin (IL)-1β, IL-6, IL-8, tumor necrosis factor alpha (TNF-α), chemokine, interferons] and anti-inflammatory cytokines (e.g., IL-4, IL-6, IL-10, and IL-13) play significant roles in the inflammatory responses to infections. Many studies have demonstrated the key role of cytokines in the occurrence, development, and prevalence of infectious diseases (15–17).

The study of cytokine expression characteristics in *S. hyicus* infection is significant for understanding the inflammatory response and the pathogenic mechanism of *S. hyicus* and may facilitate efficient treatment in *S. hyicus* infection. To date, there are no reports on the cytokine response in piglets infected with *S. hyicus*. Thus, in the present study, we investigated the expression of 10 serum cytokines [IL-1β, IL-4, IL-6, IL-8, IL-10, IL-12, granulocyte-macrophage colony-stimulating factor (GM-CSF), interferon (IFN)-γ, transforming growth factor (TGF)-β1, and TNF-α] in piglets infected with toxigenic and non-toxigenic *S. hyicus*.

## Materials and Methods

### Bacteria

A toxigenic *S. hyicus* strain, ZC-4 (GenBank no. JQ728535), carrying exfoliative toxin ExhA was isolated in Guangdong Province, China, in 2010. A non-toxigenic *S. hyicus* strain, CF-1 (GenBank no. JQ728492), carrying no exfoliative toxin was isolated in Guangdong Province, China, in 2011.

### Animals and Experimental Design

Fifteen, 25-day-old, *S. hyicus*-free piglets were obtained from a farm in Guangzhou and randomly divided into three groups with five piglets per group. The piglets in the toxigenic and non-toxigenic groups were intramuscularly injected in the neck with *S. hyicus* ZC-4 and CF-1, respectively, at a dose of 9.0 × 10^9^ CFU per piglet. The piglets in the control group were intramuscularly injected with 2 mL of phosphate buffered saline (PBS). The animals were kept in separate rooms.

All animal procedures were approved by the Ethics Committee of Institute of Animal Health, Guangdong Academy of Agricultural Sciences according to Guangdong Province Laboratory Animal Management Regulations. The license number was SYXK (Yue) 2016-0165.

### Clinical and Pathological Examination

The piglets were monitored at 6, 24, 48, and 72 h and 1 week post inoculation for clinical signs, including rectal temperature, and any clinical signs were scored. At 6, 24, 48, and 72 h after inoculation, blood and serum samples were collected for cytokine analysis. For histological examination, samples of lung, lymph nodes, spleen, kidney, liver, and skin were collected at 72 h post inoculation, fixed in 10% formalin solution, routinely processed, and embedded in paraffin wax; tissue sections were stained with hematoxylin and eosin (HE) as previously described. Moreover, heart and lung samples of the inoculated piglets were collected for the examination of bacterial presence using gram staining, biochemical properties and PCR amplification of the 16S rRNA gene in the toxigenic and non-toxigenic groups and *ExhA* gene in the toxigenic group.

### Bacterial Isolation, Morphology, and Biochemical Characteristics

Heart and lung samples of the infected piglets were collected aseptically for bacterial isolation and cultured on LB agar plates containing 5 % sheep red blood cells at 37°C for 18 h. Gram staining was performed using the following protocol: a single colony was placed in a drop of physiological saline on a glass slide, and the specimen was fixed carefully by passing the slide through a flame. Then, the slide was flooded with ammonium oxalate crystal violet for 1 min and then washed with water. The slide was flooded with iodine solution for 1 min followed by flooding with ethanol for 30 s. The slide was treated with sand yellow solution for 1 min, washed with water, and then air-dried. Finally, the slide was observed under the light microscope. Biochemical properties were characterized using the commercial kit from Hangzhou Microbiological Reagents Limited Company (Hangzhou, China) following the manufacturer's instructions.

### PCR Amplification of 16S rRNA and Exfoliative Toxin Gene

Bacterial DNA was extracted from pure culture of the heart and lung of piglets infected and identified as *S. hyicus* using QIAGEN DNeasy Blood and Tissue Kit (Qiagen, Basel, CH) according to the manufacturer's protocol. PCR amplification of 16S rRNA and exfoliative toxin gene was performed using the following primers: 16S rRNA-F (5′-AGAGTTTGATCCTGGCTTAG-3′); 16S rRNA-R (5′-TGACGGGCGGTGTGTACAA-3′). ExhA-F (5′-ATAGAGGAGAAATCAACATG-3′); ExhA-R (5′-CTATAGTTACTTGACCTCTA -3′).

In brief, the reaction mixture (final volume 20 μL) contained 10 μL of 2×Taq Master Mix, 0.5 μM of forward and reverse primer, and 1 μL of template DNA. The thermal cycling consisted of an initial denaturation step at 94°C for 5 min; followed by 35 cycles at 94°C for 1 min, 56°C for 1 min, and 72°C for 90 s; and a final extension step at 72°C for 10 min.

### Cytokine Analysis

Serum levels of GM-CSF, IL-1β, IL-6, IL-10, TGF-β1, IFNγ, IL-4, IL-8, IL-12p40, and TNF-α in piglets were measured using the RayBiotech porcine cytokine array Q1 (QAP-CYT-1-1, RayBiotech, Norcross, GA, USA) according to the manufacturer's instructions.

### ELISA Analysis

ELISA was used to determine the serum concentration of IL-1β, IL-8, IL-10, and IL-12 in inoculated piglets. IL-1β (#ELP-IL1b-1) and IL-8 (#ELP-IL8-1) were quantified using RayBio^®^ ELISA kit (RayBiotech, Inc., Norcross, GA, USA), and IL-10 (#P1000) and IL-12 (P1240) were quantified by R&D ELISA kit (R&D Systems, USA).

### Statistical Analysis

All data were presented as means ± standard deviation (SD). The statistical analysis was conducted by using the unpaired Student's *t*-test in GraphPad Prism 5 software. Differences with a value of *p* < 0.05 were considered statistically significant.

## Results

### Clinical Signs and Gross Lesions

**Figure 1:**
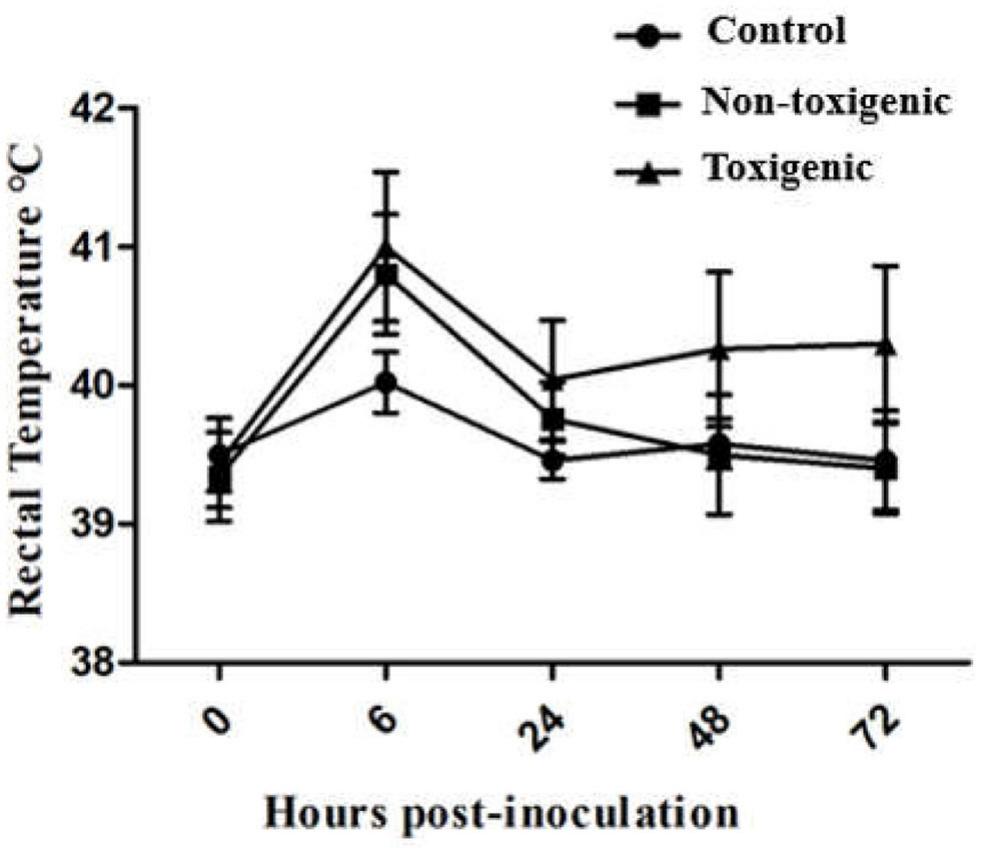
Changes in rectal temperature of piglets at 0, 6, 24, 48, and 72 h post in toxigenic group, non-toxigenic group, and control group. Each data point represents the mean ± standard deviation (SD) generated from five pigs in each group.

Piglets in the toxigenic and non-toxigenic groups showed increased rectal temperature (between 40.5 and 41°C) at 6 h post infection, but decreased rectal temperature (<40.5°C) at 24 h, 48 h, and 72 h post infection. Notably, the rectal temperature of piglets in the toxigenic group was higher than those in the non-toxigenic group at all time points (Figure 1). Piglets in the toxigenic group exhibited a range of clinical signs, such as listlessness, red body surface, trembling body, gathering, swollen eyes, and exfoliation of small areas of skin on the ears, belly, hind legs, and tails at 24 h post infection. At 48 h post infection, serious clinical signs appeared, exfoliation of large areas of skin with fluid exudation; then scabs began to form in the areas that had shed skin. Moreover, one of five piglets infected with ZC-4 died at 1 week post inoculation. In the non-toxigenic group, inoculated piglets only showed mild listlessness at 24 h post infection. Neither clinical signs (Table 1) nor significant increases in body temperature (Figure 1) were observed in control animals throughout the study. The analysis of anatomical lesions showed that, in the toxigenic group, the mandibular lymph nodes showed the most prominent gross lesions with diffused hemorrhage, whereas no significant changes were observed in lung and liver. There were no obvious pathological changes in the organs in piglets in the non-toxigenic group.

**Table 1:** Outcomes of inoculation of piglets in toxigenic and non-toxigenic groups with *Staphylococcus hyicus* infection.

**Hours post infection**	**Group**	**Clinical signs^a^**	**Morbidity^b^**	**Mortality^c^**
6 h	Control	–	0/5	0/5
	Non-toxigenic	–	0/5	0/5
	Toxigenic	–	0/5	0/5
24 h	Control	–	0/5	0/5
	Non-toxigenic	+	0/5	0/5
	Toxigenic	++	5/5	0/5
48 h	Control	–	0/5	0/5
	Non-toxigenic	–	0/5	0/5
	Toxigenic	+++	5/5	0/5
72 h	Control	–	0/5	0/5
	Non-toxigenic	–	0/5	0/5
	Toxigenic	+++	5/5	0/5
1 W	Control	–	0/4	0/4
	Non-toxigenic	–	0/4	0/4
	Toxigenic	+++	4/4	1/4

a *Clinical sign: + depressed; ++ exfoliation of a small area; +++ exfoliation of a large area; –no clinical symptom*.

b *Morbidity = Number of sick pigs/total experimental piglets per group*.

c *Mortality = Number of dead pigs/total experimental piglets per group*.

### Pathological Examination

**Figure 2:**
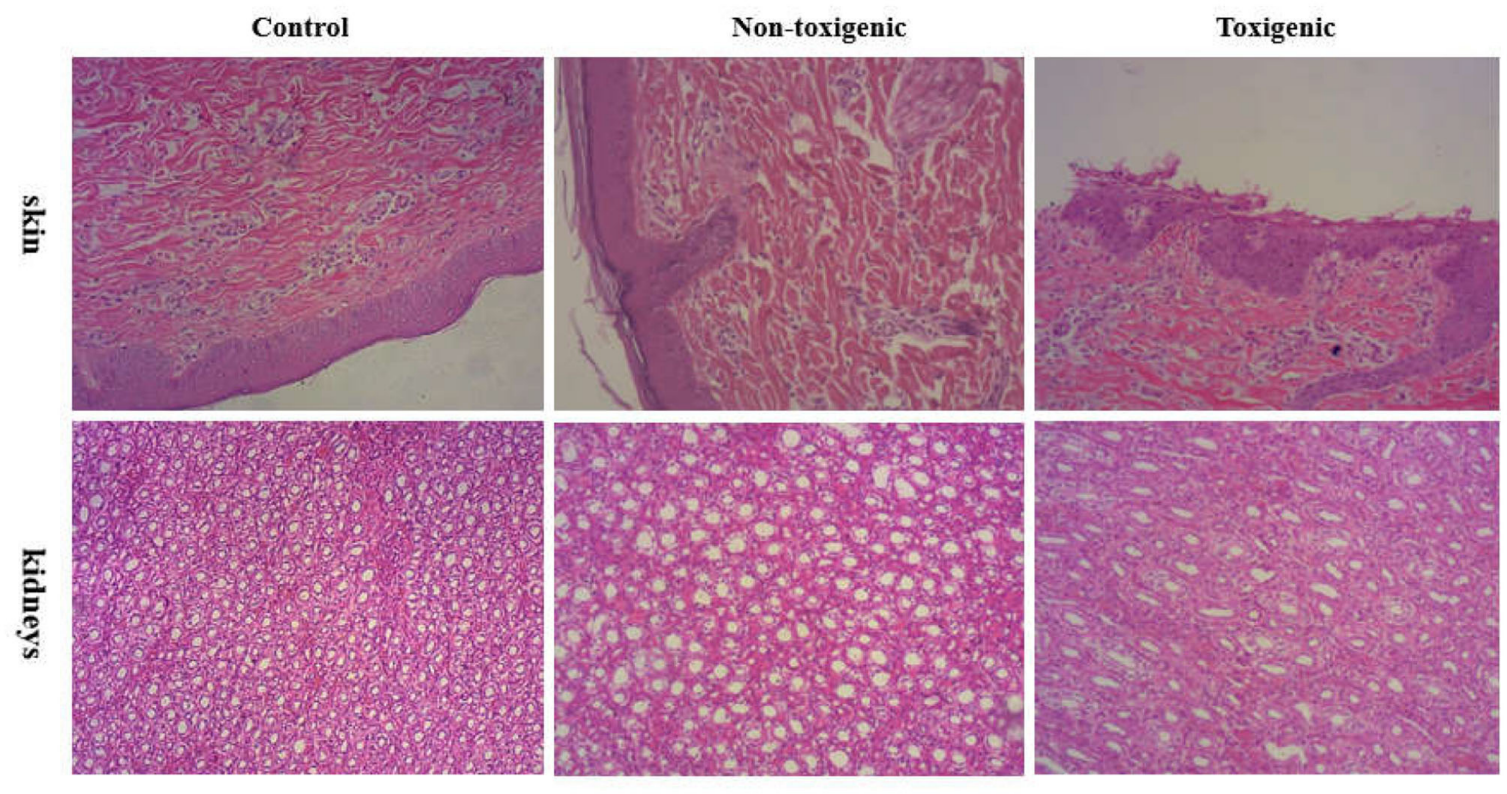
Histophathological changes in the skin and kidneys of piglets from toxigenic group, non-toxigenic group, and control group at 72 h post infection. The tissues were stained with hematoxylin and eosin (magnification 100×).

The skin and kidneys showed the most obvious lesions. Histological examination showed that the skin lesions in piglets in the toxigenic group were characterized by cuticular exfoliation, incomplete and excessive hyperkeratosis, and inflammatory cell hyperplasia of the epidermis, whereas piglets in the non-toxigenic group exhibited hyperkeratosis and inflammatory cell hyperplasia of the epidermis; the control piglets showed no pathological changes in the skin tissues. Degeneration of the uriniferous tubule epithelium was evident in the toxigenic and non-toxigenic groups, whereas no pathological changes were observed in the control group (Figure 2). The liver, lung, spleen, and lymph nodes showed no visible lesions.

### Isolation and Identification of *Staphylococcus hyicus*

*Staphylococcus* was isolated in pure culture from the heart and lung of piglets and identified as *S. hyicus* by morphological examination and biochemical identification. Gram staining showed that the isolated bacteria were clustered, grape-like cocci (*Staphylococcus*) with gram-positive staining. The results of morphological tests were confirmed by the detection of a specific fragment (1,359 bp) of 16S rRNA gene in piglets in the toxigenic and non-toxigenic groups; the fragment was not detected in the control group piglets. In addition, a specific fragment (865 bp) of the *ExhA* gene was detected in piglets in the toxigenic group but not in piglets in the non-toxigenic and control groups.

### Serum Cytokine Levels

At 6, 24, 48, and 72 h post infection, blood samples were collected, and the serum levels of 10 cytokines (GM-CSF, IL-1β, IL-6, IL-10, TGF-β1, IFNγ, IL-4, IL-8, IL-12p40, and TNF-α) were assessed. Significant differences between *S. hyicus* (CF-1 and ZC-4)-infected and control piglets and between ZC-4-infected and CF-1 infected piglets are shown in Table 2.

**Table 2:** Serum levels of cytokines in piglets over the course of *Staphylococcus hyicus* infection.

**Cytokine**	**Non-toxigenic/Control**				**Toxigenic/Control**				**Toxigenic/Non-toxigenic**			
	**6 h**	**24 h**	**48 h**	**72 h**	**6 h**	**24 h**	**48 h**	**72 h**	**6 h**	**24 h**	**48 h**	**72 h**
IL-1β	**<0.05**	ns	ns	ns	**<0.05**	ns	ns	ns	ns	ns	ns	ns
IL-4	ns	ns	ns	ns	ns	ns	ns	ns	ns	ns	ns	ns
IL-6	ns	ns	ns	ns	ns	ns	ns	ns	ns	ns	ns	ns
IL-8	ns	**<0.05**	ns	ns	**<0.05**	**<0.0001**	ns	ns	ns	**<0.05**	ns	ns
IL-10	ns	ns	ns	ns	ns	ns	**<0.05**	ns	ns	ns	**<0.05**	ns
IL-12	ns	ns	ns	ns	ns	ns	ns	**<0.05**	ns	ns	ns	**<0.01**
GM-CSF	ns	ns	ns	ns	ns	ns	ns	ns	ns	ns	ns	ns
IFNγ	ns	ns	ns	ns	ns	ns	ns	ns	ns	ns	ns	ns
TGF-β1	ns	ns	ns	ns	ns	ns	ns	ns	ns	ns	ns	ns
TNF-α	ns	ns	ns	ns	ns	ns	ns	ns	ns	ns	ns	ns

*The p-value was conducted by using the unpaired Student's t-test in GraphPad Prism 5 software. ns, not significant, differences with a value of p < 0.05, p < 0.01, and p < 0.0001 were considered statistically significant*.

**Figure 3:**
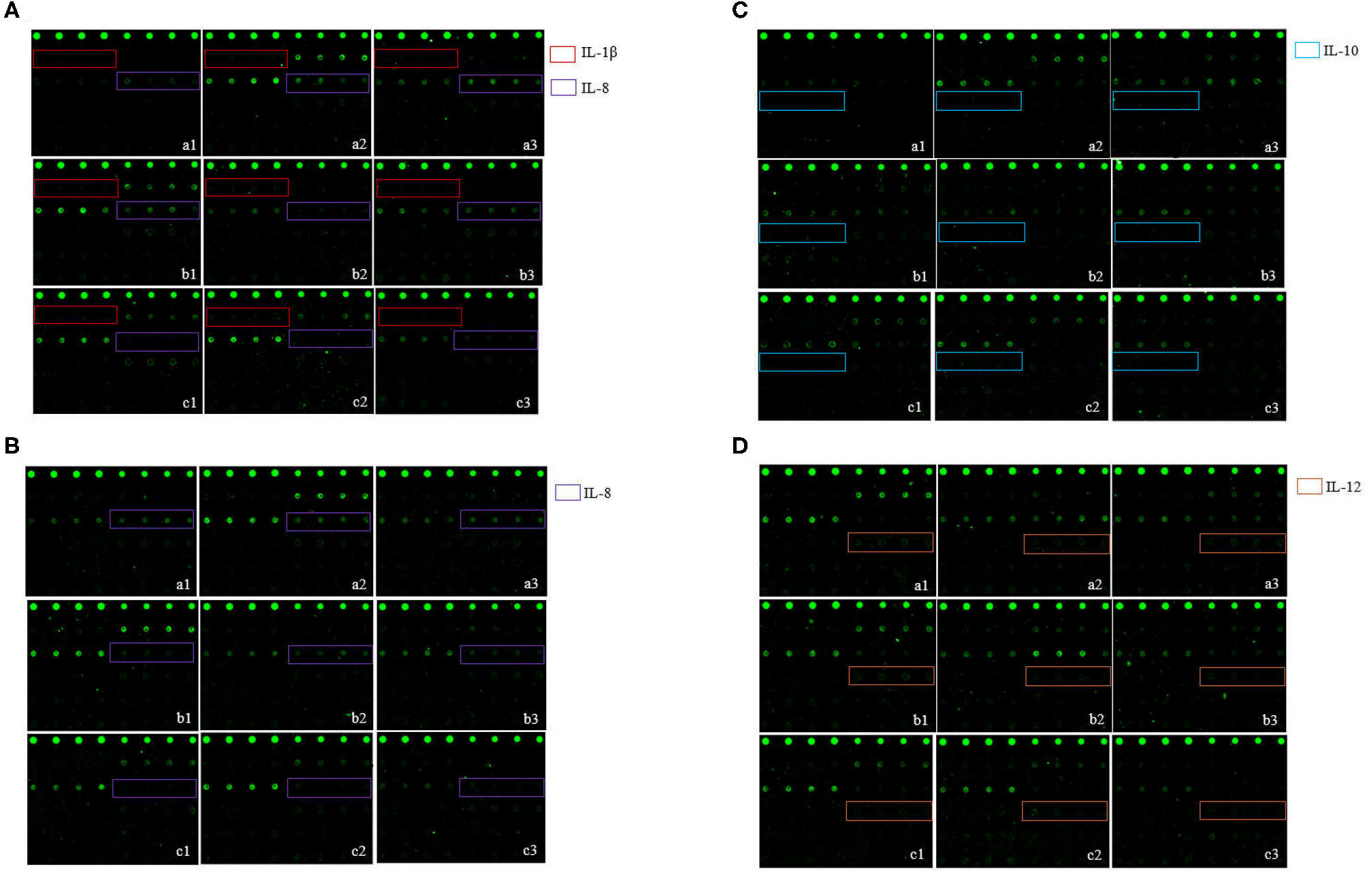
The *S. hyicus* infection associated serum cytokine profiles in piglets at 6 h **(A)**, 24 h **(B)**, 48 h **(C)**, and 72 h **(D)** post-inoculation. In the profiles of antibody arrays, the levels of cytokines are proportional to their flurescence intensity. In these arrays, each antibody was printed in four duplicates, and the locations of the serum cytokines are noted in colored boxes. a1, a2, and a3 represent the results of three different piglets in control group, b1, b2, and b3 represent the results of three different piglets in non-toxigenic group, c1, c2, and c3 represent the results of three different piglets in toxigenic group.

**Figure 4:**
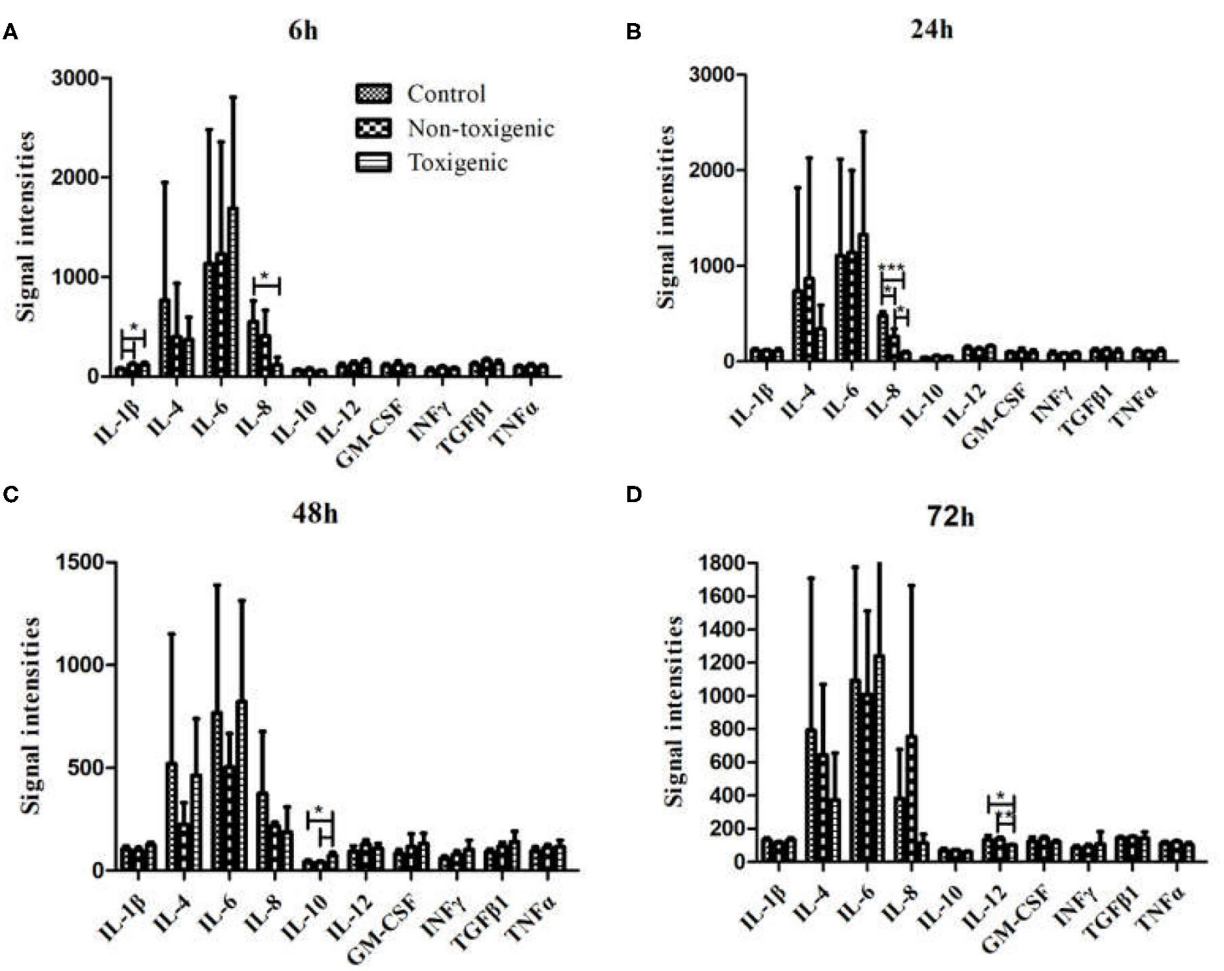
Serum cytokine levels in *Staphylococcus hyicus*-infected piglets at 6 h **(A)**, 24 h **(B)**, 48 h **(C)**, and 72 h **(D)** post inoculation. The significance of value were tested by Graphpad Software. ***p < 0.0001, **p < 0.01, *p < 0.05.

Serum levels of four cytokines (IL-1β, IL-8, IL-10, and IL-12) differed significantly between the infected and control piglets (*p* < 0.05). Among these cytokines, IL-1β levels in the toxigenic and non-toxigenic groups rapidly increased at 6 h post infection, but no significant change was observed at the following sampling time point (Figures 3A,B, 4A,B). Serum concentration of IL-8 decreased at 6 and 24 h post infection in ZC-4-infected piglets and at 24 h post infection in CF-1-infected piglets (Figures 3B, 4B). In ZC-4-infected piglets, IL-10 levels were elevated at 48 h post infection but not at other time points; in piglets in the non-toxigenic group, IL-10 levels were not elevated at any time points (Figures 3C, 4C). IL-12 levels decreased at 72 h after infection in ZC-4-infected piglets, whereas no decrease was observed in the CF-1 group (Figures 3D, 4D). Levels of three cytokines were significantly different between the toxigenic and non-toxigenic groups; IL-8 and IL-12 levels were lower in ZC-4-infected piglets at 24 and 72 h post infection, respectively, whereas IL-10 levels were higher in ZC-4-infected piglets at 48 h post infection.

### Validation of Cytokine Levels With the ELISA Assay

**Figure 5:**
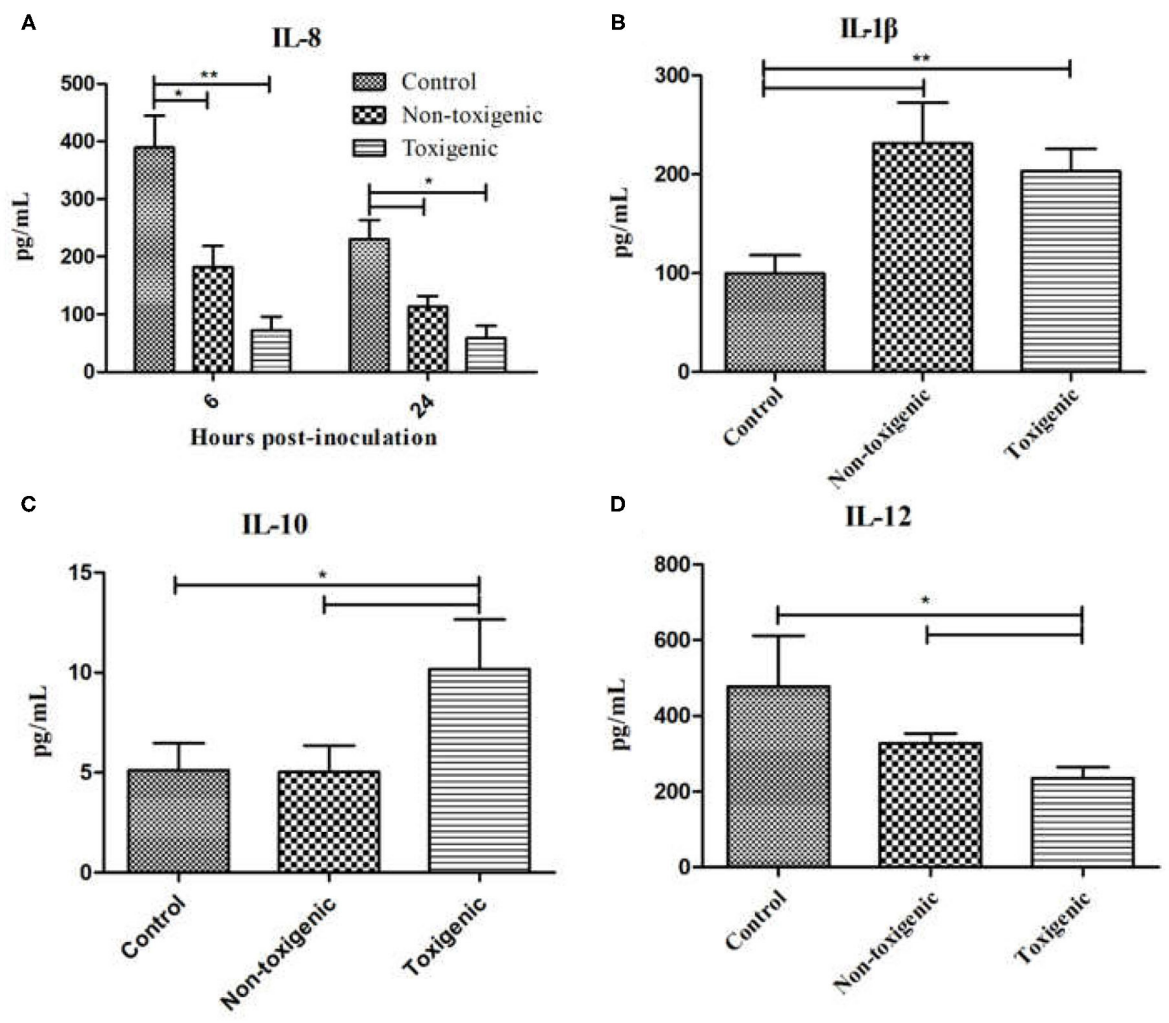
Serum interleukin (IL)-8, IL-1β, IL-10, and IL-12 levels in *Staphylococcus hyicus*-infected piglets using ELISA. Data are expressed as mean (± SD) protein concentration (pg/mL). **p < 0.01; *p < 0.05. IL-8 expression at 6 and 24 h post inoculation **(A)**, IL-1β expression at 6 h post inoculation **(B)**, IL-10 expression at 48 h post inoculation **(C)**, IL-12 expression at 72 h post inoculation **(D)**.

To confirm our observations from the cytokine antibody array, ELISA assay was used to quantitatively measure the expression levels of IL-8, IL-1β, IL-10, and IL-12. As shown in Figure 5A, IL-8 expression in the toxigenic and non-toxigenic groups was significantly lower (*p* < 0.05) than that in the control group at 6 and 24 h after infection. In the toxigenic and non-toxigenic groups, IL-1β levels were significantly higher (*p* < 0.05) than those in the control group (Figure 5B). Notably, 48 h post infection, IL-10 levels in the toxigenic group were significantly higher (*p* < 0.05) than those in the non-toxigenic and control groups; IL-10 levels in the non-toxigenic group and control groups did not differ significantly (Figure 5C). IL-12 levels in the toxigenic group were significantly lower than those in the non-toxigenic and control groups; there was no significant difference in the IL-12 levels between the non-toxigenic and control groups (Figure 5D).

## Discussion

The cytokine network plays a critical role in modulating the inflammatory response to bacterial infection (18, 19). The host immune response is a significant predictor of both persistence of infection and outcomes (20–22). To date, the host immune response, especially cytokine response, induced by *S. hyicus* remains poorly understood; therefore, more research is required to better characterize it. In the present study, we evaluated the inflammatory response in piglets infected with toxigenic and non-toxigenic strains of *S. hyicus* by measuring the serum levels of cytokines, including pro-inflammatory cytokines (IL-1β, IL-6, IL-8, TNF-α, and IFNγ) and anti-inflammatory cytokines (IL-4, IL-10, and TGF-β1).

The results demonstrated that piglets infected with toxigenic and non-toxigenic *S. hyicus* strains exhibited differential clinical signs and outcomes; the morbidity and mortality rates were higher in the toxigenic group than in the non-toxigenic group and control group, indicating the stronger virulence of the toxigenic strain ZC-4 than that of non-toxigenic strain CF-1. These results are consistent with previous reports that the exfoliative toxin is the most important determinant of *S. hyicus* virulence and may cause dehydration and subsequent death in piglets (8–10). Infection with the toxigenic strain of *S. hyicus* induced both an inflammatory reaction and an anti-inflammatory response as indicated by the cytokine assay results—a significant increase in the levels of a pro-inflammatory cytokine (IL-1β) and an anti-inflammatory cytokine (IL-10). In the non-toxigenic group, only an inflammatory reaction was induced as suggested the increased serum level of IL-1β. These results demonstrated that the toxigenic and non-toxigenic strains of *S. hyicus* induce different inflammatory reactions and that the exfoliative toxin plays an important role in the inflammatory response of piglets to *S. hyicus* infection.

Pro-inflammatory cytokines such as IL-1β, IL-6, IL-8, and TNF-α mediate the early inflammatory response and amplify the inflammatory response (23, 24). In the present study, infection with *S. hyicus* (toxigenic strain and non-toxigenic strain) induced a robust IL-1β response, as indicated by the elevated serum IL-1β levels 6 h post infection in both toxigenic-strain-infected piglets and non-toxigenic-strain-infected piglets. IL-1β is a typical primary pro-inflammatory cytokine that initiates an inflammatory response in the immune system (25). Elevated levels of circulating IL-1β have been reported to cause acute inflammatory response in bacterial infection. Our findings of increased IL-1β levels were consistent with those observed in infections caused by *S. aureus and S. epidermidis* (26, 27). The serum levels of IL-8 in piglets infected with *S. hyicus* were also examined in the present study. IL-8 is another important inflammatory cytokine playing a key role in initiating the inflammatory responses against bacterial pathogens (28, 29). However, in the present study, the expression level of the inflammatory factor IL-8 was sharply decreased in both toxigenic group and non-toxigenic group at 6 and 24 h post infection, which is inconsistent with the levels induced by *S. aureus* infection (30).

IL-12 is necessary for survival in respiratory infection with methicillin-resistant *S. aureus* and increased pulmonary clearance of methicillin-resistant *S. aureus* (31). The expression level of IL-12 significantly reduced in toxigenic-strain-infected piglets 72 h post infection, consistent with our previous findings of reduced IL-12 levels in toxigenic-strain–infected mice (32) and findings of another study which also reported a lack of inflammatory IL-12 in *S. epidermidis* infection (33). IL-12 plays a key role in the development and augmentation of Th1 responses during infection and inflammation (34) and has been identified as a critical cytokine in the pathogenesis of several inflammatory diseases such as rheumatoid arthritis (35), inflammatory bowel disease (36), and *S. aureus* infection (37, 38). Notably, the IL-12 serum concentration in piglets infected with the non-toxigenic strain was not only reduced but elevated. The severe clinical signs and the reduced IL-12 levels in toxigenic-strain-infected piglets suggest that IL-12 is an important predictive factor of the infection status of piglets infected with *S. hyicus*.

Anti-inflammatory cytokines, including IL-4, IL-10, and TGF-β, regulate the inflammatory response. In addition to the pro-inflammatory response described above, a robust anti-inflammatory response was induced by the toxigenic strain of *S. hyicus*, as indicated by the increased levels of IL-10. IL-10 is a major immunomodulatory cytokine that is able to inhibit the synthesis and release of other cytokines, thereby inhibiting cell-mediated immunity and extending the duration of viremia during the early stage of infection (39, 40). Remarkably, in the present study, 48 h post infection, IL-10 levels significantly increased in the toxigenic-strain-infected piglets but not in the non-toxigenic-strain-infected piglets. Overexpression of anti-inflammatory mediators results in immunosuppression, which hinders the host's ability to clear the primary infection, and leads to the development of secondary infection (20). Induction of IL-10 production by *S. aureus* may also facilitate immune evasion, which under certain circumstances is a form of immunomodulation (41). The IL-10 data above indicates that the toxigenic strain of *S. hyicus* induces an immunosuppression response in piglets and IL-10 release is a part of the pathogenic mechanism of *S. hyicus*. The present findings of upregulated IL-10 levels are consistent with a previous report that elevated IL-10 concentration was associated with a predominantly anti-inflammatory response and worse outcome (20, 40, 42). An initial elevation of IL-10 was also found to be associated with poor outcomes in other infections, such as Bartonella Quintana bacteremia (43) and candidemia (44). In the present study, elevated serum IL-10 also predicted worse outcome, as indicated by the severe clinical signs and high mortality in the toxigenic-strain-infected piglets and the absence of clinical signs and mortality in the non-toxigenic-strain-infected piglets, which did not exhibit elevated IL-10. Previous studies have reported that *S. epidermidis* infection enhanced IL-10 expression and improved the anti-inflammatory effects (33). The present study demonstrated that exposure to *S. hyicus* increased serum IL-10 levels in piglets, suggesting that IL-10 participates in the anti-inflammatory response during *S. hyicus* infection. In the present study, increased serum levels of IL-10 and decreased serum levels of IL-12 were both observed in the toxigenic-strain-infected piglets, which was consistent with the findings in *S. epidermidis* infection (33).

A previous study indicated that *S. aureus* exotoxins contribute to evading the host immune response and destroying host tissue, thus increasing the severity of infection (45). In the present study, the *S. hyicus* strain ZC-4 with exfoliative toxin ExhA induced a strong anti-inflammatory response and increased the severity of infection; thus, we speculated that the exfoliative toxin of *S. hyicus* is a potential key factor contributing to the anti-inflammatory response. Several studies have characterized the host inflammatory responses induced by *S. aureus* and *S. epidermidis* (46, 47), but to date, no research on the inflammatory response induced by *S. hyicus* has been reported. This study is the first to evaluate the host cytokine response to *S. hyicus* infection in piglets, contributing to the knowledge on pathogenesis of *S. hyicus* and providing basic information for treating exudative epidermitis (EE) in pigs.

This is a preliminary study about the host cytokine response induced by *S. hyicus*, and there are several limitations to our study. Because only one toxigenic strain and one non-toxigenic strain were used to study the cytokine response, data obtained are insufficient to comprehensively analyze the inflammatory response induced by *S. hyicus*. More toxigenic strains should be used to investigate the levels of anti-inflammatory cytokines to verify the findings on the anti-inflammatory response and cytokines critical for the pathogenicity of a toxigenic strain of *S. hyicus*.

## Conclusions

In conclusion, the present study found that the pro-inflammatory cytokine IL-1β was upregulated in piglets infected with a toxigenic strain and a non-toxigenic strain of *S. hyicus*, and levels of the anti-inflammatory cytokine IL-10 increased only in piglets infected with the toxigenic strain; thus, the two *S. hyicus* strains induce different cytokine responses in infected piglets. The upregulated IL-1β level indicated a significant pro-inflammatory response in *S. hyicus* infection. IL-10 expression levels were also significantly upregulated, indicating that the anti-inflammatory response was induced by infection with the toxigenic strain. Our findings suggest that elevated IL-10 level maybe associated with severe *S. hyicus* infection, and IL-10 may be the key cytokine responsible for the pathogenic mechanisms of *S. hyicus*.

Presently, our knowledge of the ability of *S. hyicus* to induce an inflammatory response during infection is limited. Future research should include an in-depth analysis of the inflammatory response in *S. hyicus* infection using more *S. hyicus* strains and the factors, such as toxins, that contribute to inflammation and immune priming.

## Data Availability Statement

The original contributions presented in the study are included in the article/Supplementary Material, further inquiries can be directed to the corresponding author/s.

## Ethics Statement

The animal study was reviewed and approved by the Animal Experimental Ethics Committee of the Institute of Animal Health, Guangdong Academy of Agricultural Sciences.

## Author Contributions

YL performed the experiments and drafted the manuscript. SS, ZB, and RC prepared materials for the experiments. PC and ZJ participated in the experiments. KZ and HG contributed to the data analysis. CL conceived the study. All authors read and approved the final manuscript.

## Conflict of Interest

The authors declare that the research was conducted in the absence of any commercial or financial relationships that could be construed as a potential conflict of interest.
