# Supplementary material for: Comparison of Host Cytokine Response in Piglets Infected With Toxigenic and Non-toxigenic Staphylococcus hyicus
Source: Front Vet Sci. 2021 Feb 16;8:639141. doi: 10.3389/fvets.2021.639141 (PMC7920954; doi:10.3389/fvets.2021.639141)
Supplement: Supplementary file 1 [file Data_Sheet_1.PDF]

1. The piglets infected with toxigenic and non-toxigenic *S. hyicus* strains showed different clinical sings.

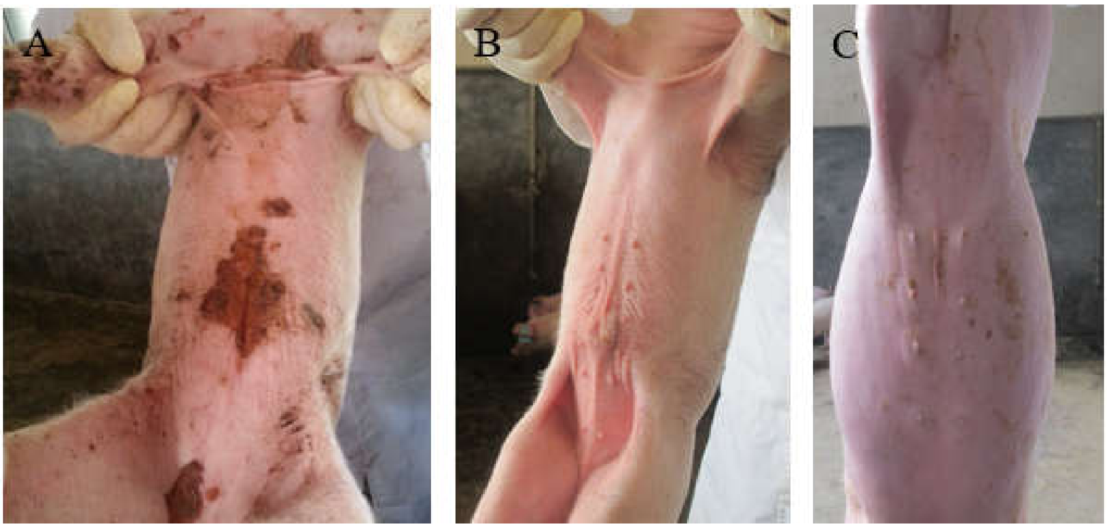

Figure. 1. The clinical sign of piglets infected with *Staphylococcus hyicus* strain ZC-4 (A), CF-1 (B), and control (C).

2. The bacterial morhology of *S. hyicus* was observed uder microscope.

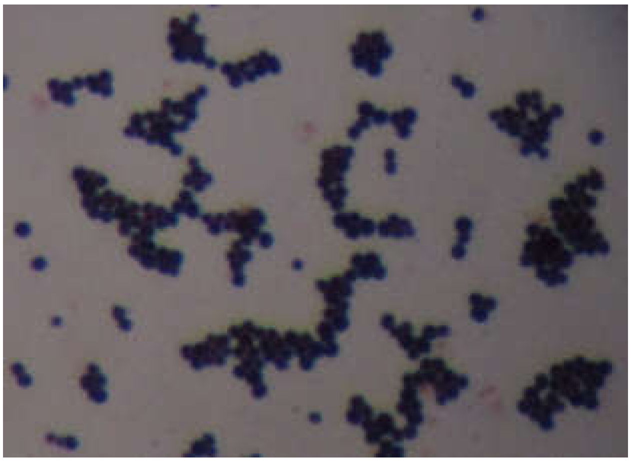

Figure.2. The results of gram staining microscopic examination (100×)

3. The 16S rRNA and exfoliative toxin gene (ExhA) genes were detected by PCR.

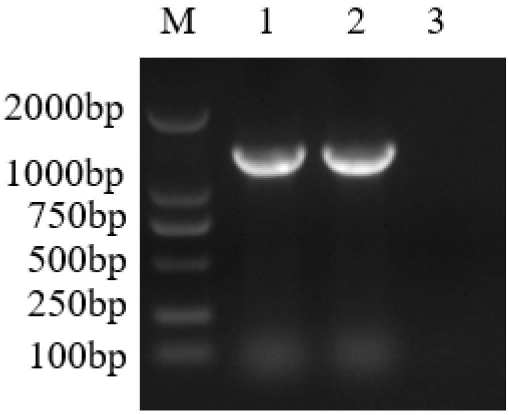

Figure.3. Identification result of 16S rRNA by PCR

M: DL2000 DNA Marker; 1: ZC-4; 2: CF-1; 3: Control

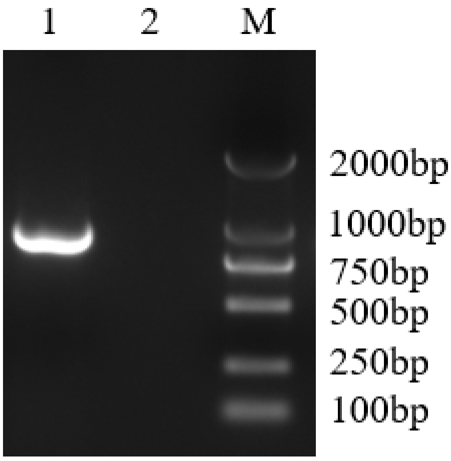

Figure.4. Identification result of ExhA by PCR

M: DL2000 DNA Marker; 1: ZC-4; 2: CF-1
